# Efficient and accurate P-value computation for Position Weight Matrices

**DOI:** 10.1186/1748-7188-2-15

**Published:** 2007-12-11

**Authors:** Hélène Touzet, Jean-Stéphane Varré

**Affiliations:** 1 LIFL, UMR CNRS 8022, Université des Sciences et Technologies de Lille, 59655 Villeneuve d'Ascq, France; 2 INRIA, 40 avenue Halley, 59650 Villeneuve d'Ascq, France

## Abstract

**Background:**

Position Weight Matrices (PWMs) are probabilistic representations of signals in sequences. They are widely used to model approximate patterns in DNA or in protein sequences. The usage of PWMs needs as a prerequisite to knowing the statistical significance of a word according to its score. This is done by defining the P-value of a score, which is the probability that the background model can achieve a score larger than or equal to the observed value. This gives rise to the following problem: Given a P-value, find the corresponding score threshold. Existing methods rely on dynamic programming or probability generating functions. For many examples of PWMs, they fail to give accurate results in a reasonable amount of time.

**Results:**

The contribution of this paper is two fold. First, we study the theoretical complexity of the problem, and we prove that it is NP-hard. Then, we describe a novel algorithm that solves the P-value problem efficiently. The main idea is to use a series of discretized score distributions that improves the final result step by step until some convergence criterion is met. Moreover, the algorithm is capable of calculating the exact P-value without any error, even for matrices with non-integer coefficient values. The same approach is also used to devise an accurate algorithm for the reverse problem: finding the P-value for a given score. Both methods are implemented in a software called TFM-PVALUE, that is freely available.

**Conclusion:**

We have tested TFM-PVALUE on a large set of PWMs representing transcription factor binding sites. Experimental results show that it achieves better performance in terms of computational time and precision than existing tools.

## Background

A key problem in the understanding of gene regulation is the identification of transcription factor binding sites. Transcription factor binding sites are often modeled by *Position Weighted Matrices *(PWMs for short), also known as *Position Specific Scoring Matrices *(PSSMs for short), or simply *matrices*. Examples are to be found in the Jaspar [1] or Transfac [2] databases. The usage of such matrices goes with global bioinformatics strategies that help to elucidate regulation mechanisms: comparative genomics, identification of over-represented motifs, identification of correlation between binding sites, ... Similar matrix-based models also serve to represent splice sites in messenger RNAs [3] or signatures in amino acid sequences [4].

Matrices are probabilistic descriptions of approximate patterns. Given a finite alphabet Σ and a positive integer *m*, a matrix *M *is a function from Σ^*m *^to ℝ that associates a score to each word of Σ^*m*^. More precisely, it is indexed by {1,...,*m*} × Σ. Each column corresponds to a position in the motif and each row to a letter in the alphabet Σ. The coefficient *M *(*i*, *x*) gives the score at position *i *in [1, *m*] for the letter *x *in Σ. Given a string *u *in Σ^*m*^, the *score *of *M *on *u *is defined as the sum of the scores of each character symbol of *u*:

Score(u,M)=∑i=1mM(i,ui),
 MathType@MTEF@5@5@+=feaafiart1ev1aaatCvAUfKttLearuWrP9MDH5MBPbIqV92AaeXatLxBI9gBaebbnrfifHhDYfgasaacPC6xNi=xI8qiVKYPFjYdHaVhbbf9v8qqaqFr0xc9vqFj0dXdbba91qpepeI8k8fiI+fsY=rqGqVepae9pg0db9vqaiVgFr0xfr=xfr=xc9adbaqaaeGacaGaaiaabeqaaeqabiWaaaGcbaGaee4uamLaee4yamMaee4Ba8MaeeOCaiNaeeyzauMaeiikaGIaemyDauNaeiilaWIaemyta0KaeiykaKIaeyypa0ZaaabCaeaacqWGnbqtcqGGOaakcqWGPbqAcqGGSaalcqWG1bqDdaWgaaWcbaGaemyAaKgabeaakiabcMcaPaWcbaGaemyAaKMaeyypa0JaeGymaedabaGaemyBa0ganiabggHiLdGccqGGSaalaaa@48DE@

where *u*_*i *_denotes the character symbol at position *i *in *u*.

Searching for occurrences of a matrix in a sequence requires to choose an appropriate score threshold to decide whether a position is relevant or not. Let *α *be such a score. We say that the matrix *M *has an *occurrence *in the sequence *S *at position *i *if Score(*S*_*i *_... *S*_*i*+*m*-1_, *M*) ≥ *α*. The problem of efficiently finding occurrences of a matrix in a text has recently attracted a lot of interest [5-7]. Here we address the problem of computing the score threshold *α*. To determine such a score threshold, the standard method is to use a P-value function, which gives the statistical significance of an occurrence according to its score. The P-value P-value(*M*, *α*) is the probability that the background model can achieve a score equal to or greater than *α*. In other words, the P-value is the proportion of strings (with respect to the background model) whose score is greater than the threshold *α *for *M*. In [8], the authors introduce a generic approach to P-value computation for non-parametric models. In the context of matrices, the computation can be carried out using probability generating functions or dynamic programming [9-12]. In both cases, the time complexity is proportional to the product of the length of the matrix and the number of possible different scores. If the matrix has non-negative integer coefficient values, then the number of possible different scores is bounded by ∑i=1mmax⁡{M(i,x)|x∈Σ}
 MathType@MTEF@5@5@+=feaafiart1ev1aaatCvAUfKttLearuWrP9MDH5MBPbIqV92AaeXatLxBI9gBaebbnrfifHhDYfgasaacPC6xNi=xH8viVGI8Gi=hEeeu0xXdbba9frFj0xb9qqpG0dXdb9aspeI8k8fiI+fsY=rqGqVepae9pg0db9vqaiVgFr0xfr=xfr=xc9adbaqaaeGacaGaaiaabeqaaeqabiWaaaGcbaWaaabmaeaacyGGTbqBcqGGHbqycqGG4baEcqGG7bWEcqWGnbqtcqGGOaakcqWGPbqAcqGGSaalcqWG4baEcqGGPaqkcqGG8baFcqWG4baEcqGHiiIZcqqHJoWucqGG9bqFaSqaaiabdMgaPjabg2da9iabigdaXaqaaiabd2gaTbqdcqGHris5aaaa@4639@. It follows that known algorithms are pseudo-polynomial. In real life, matrices have actually real coefficient values, such as log-ratio matrices, or entropy matrices. In this context, the number of different scores that the matrix can achieve is significantly larger.

**Figure 1:**
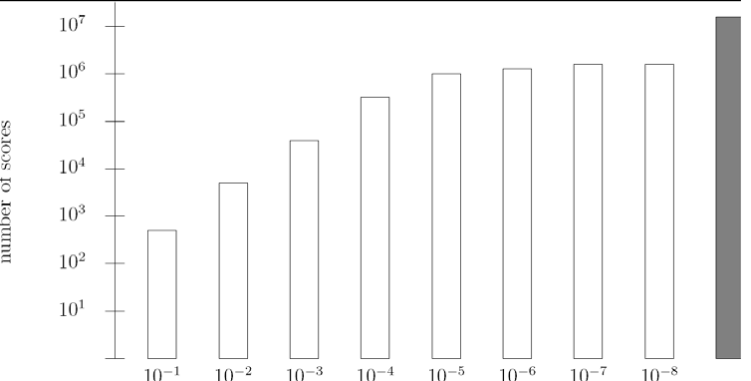
**Number of scores for a round matrix**. The matrix MA0041 of length 12 from the Jaspar database has been round with a number of digits after the decimal point from 1 to 8. The results are presented by a histogram showing the number of distinct scores that the round matrix can achieve. The number of scores is in log scale. The grey bar shows the number of distinct words (that is 4^12^).

**Figure 2:**
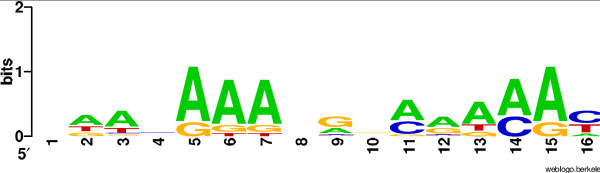
**The MA0045 Jaspar matrix logo**. The logo of the matrix MA0045 from the Jaspar database on which experiments in the Background section have been done.

Theoretically, it can be as high as |Σ|^*m*^. The usual way to deal with real matrices is to round them at a given precision, such as a given number of digits after the decimal point. In this context, the number of scores depends strongly on the chosen precision. Figure 1 displays such an example. It shows the number of distinct scores obtained with the matrix MA0041 from the Jaspar database for a variety of rounding values. With a precision set to 10^-6^, we get more than one million distinct scores. Existing algorithms have difficulties to deal with such a large number of scores. An alternative consists in using a rough estimation, such a 10^-3^. In this context, the estimated distribution induced by the round matrix is likely to give larger error rates. For example, Figure 2 shows the logo [13] of the matrix MA0045 of length 16 from the Jaspar database. We chose 5 as a score threshold, which corresponds approximately to a P-value equal to 10^-3^. The number of words whose score is greater than or equal to 5 is 4045101 onto the original matrix, compared to 4034054 for the round matrix with a precision of 10^-3^. This makes a difference of 11047 words. This error naturally affects the accuracy to the P-value. To estimate this, we conducted a large scale experiment on all Jaspar matrices (123 matrices) for a variety of precisions and a uniform P-value set to 10^-3^. We compared the number of words whose score is larger than the threshold when the P-value is computed from the corresponding round matrix to the correct number of words that is observed with the true matrix, without discretization. In each case, we indicate the percentage of matrices for which the number of words is different. Results are reported in Table 1. With a rounding at the third digits after the decimal point, 55 percent of matrices give false results. Even with a rounding at the sixth digits after the decimal point, there exist matrices for which the discretization gives a false result. This demonstrates that it may be necessary to use high precision scores to obtain accurrate results. The choice of the precision is a difficult compromise between accuracy and tractability. To the best of our knowledge, this question is passed over in silence by existing algorithms.

**Table 1:** Error with round matrices. We report the percentage of Jaspar matrices for which the P-value computed from a round matrix leads to a different number of words as for the P-value computed with the original matrix. The rounding ranges from 10^-2 ^to 10^-6^, and the P-value is 10^-3 ^for a multinomial background model.

Granularity	10^-2^	10^-3^	10^-4^	10^-5^	10^-6^
% matrices with error	76	55	30	15	7

In this paper, we study the theoretical complexity of the P-value problem and prove that it is intrinsically difficult. It is actually NP-hard. We then introduce a novel algorithm that achieves significant speed up compared to existing algorithms when we allow for some errors like other methods do. This algorithm is also capable to solve the P-value problem without error within a reasonable amount of time.

## Complexity of the P-value problem

We begin by introducing formally the P-value problem. We actually define two complementary problems, depending on what is given and what is searched for. In both cases, we are given a finite alphabet Σ, a matrix *M *of length *m *and a probability distribution on Σ^*m*^. We say that *s *in ℝ is an *accessible score *if there exists a word *u *in Σ^*m *^such that Score(*u*, *M*) = *s*.

*P-value problem – from score to P-value: *Given a score value *α*, find the probability of the set {*u *∈ Σ^*m*^, Score(*u*, *M*) ≥ *α*}. This probability is denoted P-value(*M*, *α*).

*Threshold problem – from P-value to score: *Given a P-value *P *(0 ≤ *P *≤ 1), find the highest accessible score *α *such that P-value(*M*, *α*) ≥ *P*. We write Threshold(*M*, *P*) for *α*.

As we will see later on in this paper, they are closely related problems. We show here that neither of them admits a polynomial algorithm, unless P = NP. For that, we first define the decision problem ACCESSIBLE SCORE as follows.

*Instance: *a finite alphabet Σ, a matrix *M *of length *m *whose coefficients are natural numbers, a natural number *t*

*Question: *does there exist a string *u *of Σ^*m *^such that Score(*u*, *M*) = *t*?

**Theorem 1 **ACCESSIBLE SCORE *is NP-hard*.

The proof of Theorem 1 is by reduction of the SUBSET SUM problem, which is a pseudo-polynomial NP-complete problem [14].

*Instance: *a set of positive integers *A *= {*a*_0_,...,*a*_*n*_} and a positive integer *s*

*Question: *does there exist a subset *A' *of *A *such that the sum of the elements of *A' *equals exactly *s*?

**Lemma 1 ***There exists a polynomial reduction from the *SUBSET SUM *problem to the *ACCESSIBLE SCORE *problem*.

**Proof**. Let *A *= {*a*_0_,...,*a*_*n*_} be a set of positive integers, and let *s *be the target integer. We define the matrix *M *of length *n *+ 1 on the two letter alphabet Σ = {*x*, *y*} as follows: *M *(*i*, *x*) = *a*_*i *_and *M *(*i*, *y*) = 0 for each *i*, 0 ≤ *i *≤ *n*. The set *A *has 2^*n*+1 ^different subsets. So we can define a bijection *φ *from the set of subsets of *A *onto Σ^*n*+1^. For each subset *A'*, the word *φ *(*A'*) is such as the *i*th letter is *x *if and only if *a*_*i *_∈ *A'*, otherwise the *i*th letter is *y*. It is easy to see that Score(*φ *(*A'*), *M*) = *s *if, and only if, ∑_*a*∈*A' *_*a *= *s*.

It remains to prove that the ACCESSIBLE SCORE problem polynomially reduces to instances of the *From score to P-value *and *From P-value to score*. problems. We are now given a finite alphabet Σ, a matrice *M *of length *m*, and a score value *t*.

### Reduction to the *From score to P-value *problem

We assume that the probability of each non-empty word of Σ^*m *^is non null. Under this hypothesis, the ACCESSIBLE SCORE problem admits a solution if, and only if, P-value(*M*, *t*) ≠ P-value(*M*, *t *+ 1).

### Reduction to the *From P-value to score *problem

We assume that the background model for Σ* is provided with a multinomial model. In this context, all words of length *m *have the same probability: 1|Σ|m
 MathType@MTEF@5@5@+=feaafiart1ev1aaatCvAUfKttLearuWrP9MDH5MBPbIqV92AaeXatLxBI9gBaebbnrfifHhDYfgasaacPC6xNi=xH8viVGI8Gi=hEeeu0xXdbba9frFj0xb9qqpG0dXdb9aspeI8k8fiI+fsY=rqGqVepae9pg0db9vqaiVgFr0xfr=xfr=xc9adbaqaaeGacaGaaiaabeqaaeqabiWaaaGcbaqcfa4aaSaaaeaacqaIXaqmaeaadaabdaqaaiabfo6atbGaay5bSlaawIa7amaaCaaabeqaaiabd2gaTbaaaaaaaa@338C@ and all P-values are of the form k|Σ|m
 MathType@MTEF@5@5@+=feaafiart1ev1aaatCvAUfKttLearuWrP9MDH5MBPbIqV92AaeXatLxBI9gBaebbnrfifHhDYfgasaacPC6xNi=xH8viVGI8Gi=hEeeu0xXdbba9frFj0xb9qqpG0dXdb9aspeI8k8fiI+fsY=rqGqVepae9pg0db9vqaiVgFr0xfr=xfr=xc9adbaqaaeGacaGaaiaabeqaaeqabiWaaaGcbaqcfa4aaSaaaeaacqWGRbWAaeaadaabdaqaaiabfo6atbGaay5bSlaawIa7amaaCaaabeqaaiabd2gaTbaaaaaaaa@33FB@. Solving the ACCESSIBLE SCORE problem amounts to decide whether there exists an integer *k*, 0 ≤ *k *≤ |Σ|^*m*^, such that Threshold(*M*, k|Σ|m
 MathType@MTEF@5@5@+=feaafiart1ev1aaatCvAUfKttLearuWrP9MDH5MBPbIqV92AaeXatLxBI9gBaebbnrfifHhDYfgasaacPC6xNi=xH8viVGI8Gi=hEeeu0xXdbba9frFj0xb9qqpG0dXdb9aspeI8k8fiI+fsY=rqGqVepae9pg0db9vqaiVgFr0xfr=xfr=xc9adbaqaaeGacaGaaiaabeqaaeqabiWaaaGcbaqcfa4aaSaaaeaacqWGRbWAaeaadaabdaqaaiabfo6atbGaay5bSlaawIa7amaaCaaabeqaaiabd2gaTbaaaaaaaa@33FB@) = *t*. The existence of such *k *can be decided with iterative computations of *From P-value to Score *for different values of *k*. This search can be performed within *O*(log_2 _(|Σ|^*m*^)) steps using binary search, because *k *decreases monotonically in *t *and there are at most |Σ|^*m *^different values for *k*.

## Algorithms for the P-value problems

From now on, we assume that the positions in the sequence are independently distributed. We denote *p*(*x*) the background probability associated to the letter *x *of the alphabet Σ. By extension, we write *p*(*u*) for the probability of the word *u *= *u*_1 _... *u*_*m*_: *p*(*u*) = *p*(*u*_1_) × ⋯ × *p*(*u*_*m*_).

### Definition of the score distribution

The computation of the P-value is done through the computation of the *score distribution*. This concept is the core of the large majority of existing algorithms [9-11,15]. Given a matrix *M *of length *m *and a score *α*, we define *Q*(*M*, *α*) as the probability that the background model can achieve a score equal to *α*. In other words, *Q *(*M*, *α*) is the probability of the set {*u *∈ Σ^*m *^| Score(*u*, *M*) = *α*}. In the case where *s *is not an accessible score, then *Q*(*M*, *s*) = 0.

The computation of *Q *is easily performed by dynamic programming. For that purpose, we need some preliminary notation. Given two integers *i*, *j *satisfying 0 ≤ *i, j *≤ *m*, *M *[*i*..*j*] denotes the submatrix of *M *obtained by selecting only columns from *i *to *j *for all character symbols. *M *[*i*..*j*] is called a *slice *of *M*. By convention, if *i *> *j*, then *M *[*i*..*j*] is an empty matrix.

The score distribution for the slice *M *[1..*i*] is expressed from the sore distribution of the previous slice *M *[1..*i *- 1] as follows.

Q(M[1..0],s)={1if s=00otherwiseQ(M[1..i],s)=∑x∈ΣQ(M[1..i−1],s−M(i,x))×p(x)
 MathType@MTEF@5@5@+=feaafiart1ev1aaatCvAUfKttLearuWrP9MDH5MBPbIqV92AaeXatLxBI9gBaebbnrfifHhDYfgasaacPC6xNi=xI8qiVKYPFjYdHaVhbbf9v8qqaqFr0xc9vqFj0dXdbba91qpepeI8k8fiI+fsY=rqGqVepae9pg0db9vqaiVgFr0xfr=xfr=xc9adbaqaaeGacaGaaiaabeqaaeqabiWaaaGcbaqbaeaabiWaaaqaaiabdgfarjabcIcaOiabd2eanjabcUfaBjabigdaXiabc6caUiabc6caUiabicdaWiabc2faDjabcYcaSiabdohaZjabcMcaPaqaaiabg2da9aqaamaaceqabaqbaeaabiGaaaqaaiabigdaXaqaaiabbMgaPjabbAgaMjabbccaGiabdohaZjabg2da9iabicdaWaqaaiabicdaWaqaaiabb+gaVjabbsha0jabbIgaOjabbwgaLjabbkhaYjabbEha3jabbMgaPjabbohaZjabbwgaLbaaaiaawUhaaaqaaiabdgfarjabcIcaOiabd2eanjabcUfaBjabigdaXiabc6caUiabc6caUiabdMgaPjabc2faDjabcYcaSiabdohaZjabcMcaPaqaaiabg2da9aqaamaaqafabaGaemyuaeLaeiikaGIaemyta0Kaei4waSLaeGymaeJaeiOla4IaeiOla4IaemyAaKMaeyOeI0IaeGymaeJaeiyxa0LaeiilaWIaem4CamNaeyOeI0Iaemyta0KaeiikaGIaemyAaKMaeiilaWIaemiEaGNaeiykaKIaeiykaKIaey41aqRaemiCaaNaeiikaGIaemiEaGNaeiykaKcaleaacqWG4baEcqGHiiIZcqqHJoWuaeqaniabggHiLdaaaaaa@816B@

The time complexity is in *O*(*m*|Σ|*S*), and the space complexity in *O*(*S*), where *S *is the number of scores that have to be visited. If coefficients of *M *are natural numbers, then *S *is bounded by *m *× max {*M *(*i*, *x*) | *x *∈ Σ, 1 ≤ *i *≤ *m*}. Equation 1 enables to solve the *From score to P-value *and *From P-value to score *problems. Given a score *α*, the P-value is obtained with the relation:

P-value(M,α)=∑s≥αQ(M,s)
 MathType@MTEF@5@5@+=feaafiart1ev1aaatCvAUfKttLearuWrP9MDH5MBPbIqV92AaeXatLxBI9gBaebbnrfifHhDYfgasaacPC6xNi=xI8qiVKYPFjYdHaVhbbf9v8qqaqFr0xc9vqFj0dXdbba91qpepeI8k8fiI+fsY=rqGqVepae9pg0db9vqaiVgFr0xfr=xfr=xc9adbaqaaeGacaGaaiaabeqaaeqabiWaaaGcbaGaeeiuaaLaeeyla0IaeeODayNaeeyyaeMaeeiBaWMaeeyDauNaeeyzauMaeiikaGIaemyta0KaeiilaWccciGae8xSdeMaeiykaKIaeyypa0ZaaabuaeaacqWGrbqucqGGOaakcqWGnbqtcqGGSaalcqWGZbWCcqGGPaqkaSqaaiabdohaZjabgwMiZkab=f7aHbqab0GaeyyeIuoaaaa@48A8@

Conversely, given *P*, Threshold (*M*, *P*) is computed from *Q *by searching for the greatest accessible score until the required P-value is reached.

### Computing the score distribution for a range of scores

Formula 1 does not explicitly state which score ranges should be taken into account in intermediate steps of the calculation of *Q*. To this end, we introduce the *best score *and the *worst score *of a matrix slice.

**Definition 1 (Best and worst scores) ***Let M be a matrix. The *best score *of the slice M *[*i*..*j*] *is defined as*

BS(M[i..j])=∑k=ijmax⁡{M(k,x)|x∈Σ}
 MathType@MTEF@5@5@+=feaafiart1ev1aaatCvAUfKttLearuWrP9MDH5MBPbIqV92AaeXatLxBI9gBaebbnrfifHhDYfgasaacPC6xNi=xI8qiVKYPFjYdHaVhbbf9v8qqaqFr0xc9vqFj0dXdbba91qpepeI8k8fiI+fsY=rqGqVepae9pg0db9vqaiVgFr0xfr=xfr=xc9adbaqaaeGacaGaaiaabeqaaeqabiWaaaGcbaGaeeOqaiKaee4uamLaeiikaGIaemyta0Kaei4waSLaemyAaKMaeiOla4IaeiOla4IaemOAaOMaeiyxa0LaeiykaKIaeyypa0ZaaabCaeaacyGGTbqBcqGGHbqycqGG4baEcqGG7bWEcqWGnbqtcqGGOaakcqWGRbWAcqGGSaalcqWG4baEcqGGPaqkcqGG8baFcqWG4baEcqGHiiIZcqqHJoWucqGG9bqFaSqaaiabdUgaRjabg2da9iabdMgaPbqaaiabdQgaQbqdcqGHris5aaaa@5447@

*Similarly, the *worst score *of the slice M *[*i*..*j*] *is defined as*

WS(M[i..j])=∑k=ijmin⁡{M(k,x)|x∈Σ}
 MathType@MTEF@5@5@+=feaafiart1ev1aaatCvAUfKttLearuWrP9MDH5MBPbIqV92AaeXatLxBI9gBaebbnrfifHhDYfgasaacPC6xNi=xI8qiVKYPFjYdHaVhbbf9v8qqaqFr0xc9vqFj0dXdbba91qpepeI8k8fiI+fsY=rqGqVepae9pg0db9vqaiVgFr0xfr=xfr=xc9adbaqaaeGacaGaaiaabeqaaeqabiWaaaGcbaGaee4vaCLaee4uamLaeiikaGIaemyta0Kaei4waSLaemyAaKMaeiOla4IaeiOla4IaemOAaOMaeiyxa0LaeiykaKIaeyypa0ZaaabCaeaacyGGTbqBcqGGPbqAcqGGUbGBcqGG7bWEcqWGnbqtcqGGOaakcqWGRbWAcqGGSaalcqWG4baEcqGGPaqkcqGG8baFcqWG4baEcqGHiiIZcqqHJoWucqGG9bqFaSqaaiabdUgaRjabg2da9iabdMgaPbqaaiabdQgaQbqdcqGHris5aaaa@546D@

The notion of best scores is already present in [16], where it is used to speed up the search for occurrences of a matrix in a text. It gives rise to *look ahead scoring*. Best scores allow to stop the calculation of Score(*u*, *M*) in advance as soon as it is guaranteed that the score threshold cannot be achieved, because we know the maximal remaining score. It has been exploited in [5,6] in the same context. Here we adapt it to the score distribution problem. Let *α *and *β *be two scores such that *α *≤ *β*. If one wants to compute the score distribution *Q *for the range [*α*, *β*], then given an intermediate score *s *and a matrix position *i*, we say that *Q*(*M *[1..*i*], *s*) is *useful *if there exists a word *v *of length *m *- *i *such that *α *≤ *s *+ Score(*v*, *M *[*i *+ 1..*m*]) ≤ *β*. Lemma 2 characterizes useful intermediate scores.

**Lemma 2 ***Let M be a matrix of length m, let α and β be two score bounds defining a score range for which we want to compute the score distribution Q. Q*(*M *[1..*i*], *s*) *is useful if, and only if*,

*α *- BS(*M *[*i *+ 1..*m*]) ≤ *s *≤ *β *- WS(*M *[*i *+ 1..*m*])

**Proof**. This is a straightforward consequence of Definition 1.

**Figure 3:**
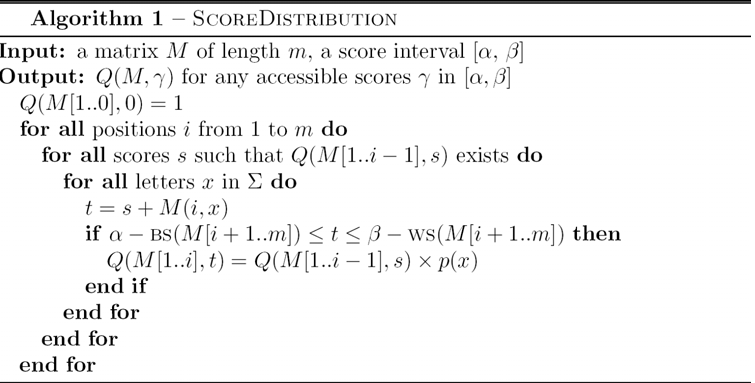
Algorithm ScoreDistribution.

This result is implemented in Algorithm SCOREDISTRIBUTION, displayed in Figure 3. The algorithm ensures that only accessible scores are visited. In practice, this is done by using a hash table for storing values of *Q*.

If one wants only to calculate the P-value of a given score without knowing the score distribution, Algorithm SCOREDISTRIBUTION can be further improved. We introduce a complementary optimization that leads to a significant speed up. The idea is that for *good *words, we can anticipate that the final score will be above the given threshold without calculating it.

**Definition 2 (Good words) ***Let α be a score and i be a position of M. Given u *= *u*_1 _... *u*_*i *_*a word of *Σ^*i*^, *we say that u is *good *for α if the following conditions are fulfilled:*

1. Score(*u*, *M *[1..*i*]) ≥ *α *- WS(*M *[*i *+ 1..*m*])

2. Score(*u*_1 _... *u*_*i*-1_, *M *[1..*i *- 1]) <*α *- WS(*M *[*i*..*m*])

**Lemma 3 ***Let u be a good word for α. Then for all v in u*Σ^*m*-|*u*|^, *we have *Score(*v, M*) ≥ *α*.

**Proof**. Let *w *in Σ^*m*-|*u*| ^such that *v *= *uw *and let *i *be the length of *u*. We have

Score(v,M)=Score(u,M[1..i])+Score(w,M[i+1..m])≥Score(u,M[1..i])+WS(M[i+1..m])≥α
 MathType@MTEF@5@5@+=feaafiart1ev1aaatCvAUfKttLearuWrP9MDH5MBPbIqV92AaeXatLxBI9gBaebbnrfifHhDYfgasaacPC6xNi=xI8qiVKYPFjYdHaVhbbf9v8qqaqFr0xc9vqFj0dXdbba91qpepeI8k8fiI+fsY=rqGqVepae9pg0db9vqaiVgFr0xfr=xfr=xc9adbaqaaeGacaGaaiaabeqaaeqabiWaaaGcbaqbaeaabmWaaaqaaiabbofatjabbogaJjabb+gaVjabbkhaYjabbwgaLjabcIcaOiabdAha2jabcYcaSiabd2eanjabcMcaPaqaaiabg2da9aqaaiabbofatjabbogaJjabb+gaVjabbkhaYjabbwgaLjabcIcaOiabdwha1jabcYcaSiabd2eanjabcUfaBjabigdaXiabc6caUiabc6caUiabdMgaPjabc2faDjabcMcaPiabgUcaRiabbofatjabbogaJjabb+gaVjabbkhaYjabbwgaLjabcIcaOiabdEha3jabcYcaSiabd2eanjabcUfaBjabdMgaPjabgUcaRiabigdaXiabc6caUiabc6caUiabd2gaTjabc2faDjabcMcaPaqaaaqaaiabgwMiZcqaaiabbofatjabbogaJjabb+gaVjabbkhaYjabbwgaLjabcIcaOiabdwha1jabcYcaSiabd2eanjabcUfaBjabigdaXiabc6caUiabc6caUiabdMgaPjabc2faDjabcMcaPiabgUcaRiabbEfaxjabbofatjabcIcaOiabd2eanjabcUfaBjabdMgaPjabgUcaRiabigdaXiabc6caUiabc6caUiabd2gaTjabc2faDjabcMcaPaqaaaqaaiabgwMiZcqaaGGaciab=f7aHbaaaaa@8752@

**Lemma 4 ***Let u be a string of *Σ^*m *^*such that *Score(*u*, *M*) ≥ *α. Then there exists a unique prefix v of u such that v is good for α*.

**Proof**. We first remark that if Score(*u*, *M*) ≥ *α*, then Score(*u*, *M*) ≥ *α *- WS(*M*[*m *+ 1..*m*]). So there exists at least one prefix of *u *satisfying the first condition of Definition 2: *u *itself. Now, consider a prefix *v *of length *i *such that Score(*v*, *M*[1..*i*]) ≥ *α *- WS(*M*[*i *+ 1..*m*]). Then for each letter *x *of Σ, we have Score(*vx*, *M*[1..*i *+ 1]) ≥ *α *- WS(*M*[*i *+ 2..*m*]): It comes from the fact that *M*(*i *+ 1, *x*) ≥ WS(*M*[*i *+ 1..*m*]) - WS(*M*[*i *+ 2..*m*]). This property implies that if a prefix *v *of *u *satisfies the first condition of Definition 2, then all longer prefixes also do. According to the second condition of Definition 2, it follows that only the shortest prefix *v *such that Score(*v*, *M*[1..*i*]) ≥ *α *- WS(*M*[*i *+ 1..*m*]) is a good word.

**Lemma 5 ***Let M be a matrix of length m*.

P-value(M,α)=∑1≤i≤m,x∈Σ,s<α−WS(M[i..m])s+M(i,x)≥α−WS(M[i+1..m])Q(M[1..i−1],s)×p(x)
 MathType@MTEF@5@5@+=feaafiart1ev1aaatCvAUfKttLearuWrP9MDH5MBPbIqV92AaeXatLxBI9gBaebbnrfifHhDYfgasaacPC6xNi=xI8qiVKYPFjYdHaVhbbf9v8qqaqFr0xc9vqFj0dXdbba91qpepeI8k8fiI+fsY=rqGqVepae9pg0db9vqaiVgFr0xfr=xfr=xc9adbaqaaeGacaGaaiaabeqaaeqabiWaaaGcbaGaeeiuaaLaeeyla0IaeeODayNaeeyyaeMaeeiBaWMaeeyDauNaeeyzauMaeiikaGIaemyta0KaeiilaWccciGae8xSdeMaeiykaKIaeyypa0ZaaabuaeaacqWGrbqucqGGOaakcqWGnbqtcqGGBbWwcqaIXaqmcqGGUaGlcqGGUaGlcqWGPbqAcqGHsislcqaIXaqmcqGGDbqxcqGGSaalcqWGZbWCcqGGPaqkcqGHxdaTcqWGWbaCcqGGOaakcqWG4baEcqGGPaqkaSqaauaabeqaceaaaeaacqaIXaqmcqGHKjYOcqWGPbqAcqGHKjYOcqWGTbqBcqGGSaalcqWG4baEcqGHiiIZcqqHJoWucqGGSaalcqWGZbWCcqGH8aapcqWFXoqycqGHsislcqqGxbWvcqqGtbWucqGGOaakcqWGnbqtcqGGBbWwcqWGPbqAcqGGUaGlcqGGUaGlcqWGTbqBcqGGDbqxcqGGPaqkaeaacqWGZbWCcqGHRaWkcqWGnbqtcqGGOaakcqWGPbqAcqGGSaalcqWG4baEcqGGPaqkcqGHLjYScqWFXoqycqGHsislcqqGxbWvcqqGtbWucqGGOaakcqWGnbqtcqGGBbWwcqWGPbqAcqGHRaWkcqaIXaqmcqGGUaGlcqGGUaGlcqWGTbqBcqGGDbqxcqGGPaqkaaaabeqdcqGHris5aaaa@8CC7@

**Proof**. We consider the set P
 MathType@MTEF@5@5@+=feaafiart1ev1aaatCvAUfKttLearuWrP9MDH5MBPbIqV92AaeXatLxBI9gBaebbnrfifHhDYfgasaacPC6xNi=xH8viVGI8Gi=hEeeu0xXdbba9frFj0xb9qqpG0dXdb9aspeI8k8fiI+fsY=rqGqVepae9pg0db9vqaiVgFr0xfr=xfr=xc9adbaqaaeGacaGaaiaabeqaaeqabiWaaaGcbaGaeeiuaafaaa@2CFA@(*α*) of words whose score is greater than or equal to *α*: P
 MathType@MTEF@5@5@+=feaafiart1ev1aaatCvAUfKttLearuWrP9MDH5MBPbIqV92AaeXatLxBI9gBaebbnrfifHhDYfgasaacPC6xNi=xH8viVGI8Gi=hEeeu0xXdbba9frFj0xb9qqpG0dXdb9aspeI8k8fiI+fsY=rqGqVepae9pg0db9vqaiVgFr0xfr=xfr=xc9adbaqaaeGacaGaaiaabeqaaeqabiWaaaGcbaGaeeiuaafaaa@2CFA@(*α*) = {*w *∈ Σ^*m*^|Score(*w*, *M*) ≥ *α*}. According to Lemma 4, each word of P
 MathType@MTEF@5@5@+=feaafiart1ev1aaatCvAUfKttLearuWrP9MDH5MBPbIqV92AaeXatLxBI9gBaebbnrfifHhDYfgasaacPC6xNi=xH8viVGI8Gi=hEeeu0xXdbba9frFj0xb9qqpG0dXdb9aspeI8k8fiI+fsY=rqGqVepae9pg0db9vqaiVgFr0xfr=xfr=xc9adbaqaaeGacaGaaiaabeqaaeqabiWaaaGcbaGaeeiuaafaaa@2CFA@(*α*) has a unique prefix that is good for *α*. Conversely, Lemma 3 ensures that each word whose prefix is good for *α *belongs to P
 MathType@MTEF@5@5@+=feaafiart1ev1aaatCvAUfKttLearuWrP9MDH5MBPbIqV92AaeXatLxBI9gBaebbnrfifHhDYfgasaacPC6xNi=xH8viVGI8Gi=hEeeu0xXdbba9frFj0xb9qqpG0dXdb9aspeI8k8fiI+fsY=rqGqVepae9pg0db9vqaiVgFr0xfr=xfr=xc9adbaqaaeGacaGaaiaabeqaaeqabiWaaaGcbaGaeeiuaafaaa@2CFA@(*α*). P
 MathType@MTEF@5@5@+=feaafiart1ev1aaatCvAUfKttLearuWrP9MDH5MBPbIqV92AaeXatLxBI9gBaebbnrfifHhDYfgasaacPC6xNi=xH8viVGI8Gi=hEeeu0xXdbba9frFj0xb9qqpG0dXdb9aspeI8k8fiI+fsY=rqGqVepae9pg0db9vqaiVgFr0xfr=xfr=xc9adbaqaaeGacaGaaiaabeqaaeqabiWaaaGcbaGaeeiuaafaaa@2CFA@(*α*) can thus be expressed as a union of disjoint sets.

P(α)=∪u is good for αuΣm−|u|
 MathType@MTEF@5@5@+=feaafiart1ev1aaatCvAUfKttLearuWrP9MDH5MBPbIqV92AaeXatLxBI9gBaebbnrfifHhDYfgasaacPC6xNi=xI8qiVKYPFjYdHaVhbbf9v8qqaqFr0xc9vqFj0dXdbba91qpepeI8k8fiI+fsY=rqGqVepae9pg0db9vqaiVgFr0xfr=xfr=xc9adbaqaaeGacaGaaiaabeqaaeqabiWaaaGcbaGaeeiuaaLaeiikaGccciGae8xSdeMaeiykaKIaeyypa0ZaambuaeaacqWG1bqDcqqHJoWudaahaaWcbeqaaiabd2gaTjabgkHiTmaaemaabaGaemyDauhacaGLhWUaayjcSdaaaaqaaiabdwha1jabbccaGiabbMgaPjabbohaZjabbccaGiabbEgaNjabb+gaVjabb+gaVjabbsgaKjabbccaGiabbAgaMjabb+gaVjabbkhaYjabbccaGiab=f7aHbqab0GaeSOkIufaaaa@4FD8@

It follows that

P-value(M,α)=∑u is good for αp(u)
 MathType@MTEF@5@5@+=feaafiart1ev1aaatCvAUfKttLearuWrP9MDH5MBPbIqV92AaeXatLxBI9gBaebbnrfifHhDYfgasaacPC6xNi=xI8qiVKYPFjYdHaVhbbf9v8qqaqFr0xc9vqFj0dXdbba91qpepeI8k8fiI+fsY=rqGqVepae9pg0db9vqaiVgFr0xfr=xfr=xc9adbaqaaeGacaGaaiaabeqaaeqabiWaaaGcbaGaeeiuaaLaeeyla0IaeeODayNaeeyyaeMaeeiBaWMaeeyDauNaeeyzauMaeiikaGIaemyta0KaeiilaWccciGae8xSdeMaeiykaKIaeyypa0ZaaabuaeaacqWGWbaCcqGGOaakcqWG1bqDcqGGPaqkaSqaaiabdwha1jabbccaGiabbMgaPjabbohaZjabbccaGiabbEgaNjabb+gaVjabb+gaVjabbsgaKjabbccaGiabbAgaMjabb+gaVjabbkhaYjabbccaGiab=f7aHbqab0GaeyyeIuoaaaa@5498@

where *p*(*u*) denotes the probability of the string *u *in the background model. By definition of *Q*, we can deduce the expected result from Formula 3.

**Figure 4:**
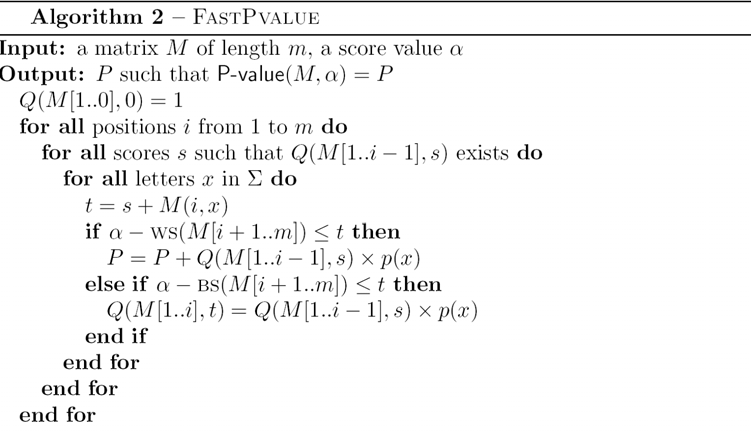
Algorithm FastPvalue.

Lemma 5 shows that it is not necessary to build the entire dynamic programming table for *Q*. Only values for *Q*(*M*[1..*i*], *s*) such that *s *<*α *- WS(*M*[*i *+ 1..*m*]) are to be computed. This gives rise to the FASTPVALUE algorithm, described in Figure 4.

### Permuting columns of the matrix

Algorithms 1 and 2 can also be used in combination with *permutated lookahead scoring *[16]. The matrix *M *can be transformed by permuting columns without modifying the overall score distribution. This is possible because the columns of the matrix are supposed to be independent. We show that it is also relevant for P-value calculation.

**Lemma 6 ***Let M and N be two matrices of length m such that there exists a permutation π on *{1,..., *m*} *satisfying, for each letter x of *Σ, *M*(*i*, *x*) = *N*(*π*_*i*_, *x*)*. Then for any α*, *Q*(*M*, *α*) = *Q*(*N*, *α*).

**Proof**. Let *u *be a word of Σ^*m *^and let v=uπ1...uπm
 MathType@MTEF@5@5@+=feaafiart1ev1aaatCvAUfKttLearuWrP9MDH5MBPbIqV92AaeXatLxBI9gBaebbnrfifHhDYfgasaacPC6xNi=xH8viVGI8Gi=hEeeu0xXdbba9frFj0xb9qqpG0dXdb9aspeI8k8fiI+fsY=rqGqVepae9pg0db9vqaiVgFr0xfr=xfr=xc9adbaqaaeGacaGaaiaabeqaaeqabiWaaaGcbaGaemODayNaeyypa0JaemyDau3aaSbaaSqaaGGaciab=b8aWnaaBaaameaacqaIXaqmaeqaaaWcbeaakiabc6caUiabc6caUiabc6caUiabdwha1jab=b8aWnaaBaaaleaacqWGTbqBaeqaaaaa@3A49@. By construction of *N*, we have Score(*u*, *M*) = Score(*v*, *N*). Since the background model is multinomial, we have *p*(*u*) = *p*(*v*). This completes the proof.

The question is how to permute the columns of a given matrix to enhance the performances of the algorithms. In [6], it is suggested to sort columns by decreasing information content. We refine this rule of thumb and propose to minimize the total size of all score ranges involved in the dynamic programming decomposition for *Q *in Algorithm SCOREDISTRIBUTION. For each *i*, 1 ≤ *i *≤ *m*, define *δ*_*i *_as *δ*_*i *_= BS(*M*[*i*..*i*]) - WS(*M*[*i*..*i*]).

**Lemma 7 ***Let M be a matrix such that δ*_1 _≥ ... ≥ *δ*_*m*_. *Then M minimizes the total size of all score ranges amongst all matrices that can be obtained by permutation of M*.

**Proof**. We write *SR*(*M*) for the total size of all score ranges of the matrix *M*. We have

SR(M)=∑i=1m−1(β−WS(M[i+1..m])−(α−BS(M[i+1..m]))+(β−α)=m(β−α)+∑i=2mBS(M[i..m])−WS(M[i..m])=m(β−α)+∑i=2m∑j=imδj=m(β−α)+∑i=2m(i−1)δi
 MathType@MTEF@5@5@+=feaafiart1ev1aaatCvAUfKttLearuWrP9MDH5MBPbIqV92AaeXatLxBI9gBaebbnrfifHhDYfgasaacPC6xNi=xI8qiVKYPFjYdHaVhbbf9v8qqaqFr0xc9vqFj0dXdbba91qpepeI8k8fiI+fsY=rqGqVepae9pg0db9vqaiVgFr0xfr=xfr=xc9adbaqaaeGacaGaaiaabeqaaeqabiWaaaGcbaqbaeaabqWaaaaabaGaem4uamLaemOuaiLaeiikaGIaemyta0KaeiykaKcabaGaeyypa0dabaWaaabmaeaacqGGOaakiiGacqWFYoGycqGHsislcqqGxbWvcqqGtbWucqGGOaakcqWGnbqtcqGGBbWwcqWGPbqAcqGHRaWkcqaIXaqmcqGGUaGlcqGGUaGlcqWGTbqBcqGGDbqxcqGGPaqkcqGHsislcqGGOaakcqWFXoqycqGHsislcqqGcbGqcqqGtbWucqGGOaakcqWGnbqtcqGGBbWwcqWGPbqAcqGHRaWkcqaIXaqmcqGGUaGlcqGGUaGlcqWGTbqBcqGGDbqxcqGGPaqkcqGGPaqkcqGHRaWkcqGGOaakcqWFYoGycqGHsislcqWFXoqycqGGPaqkaSqaaiabdMgaPjabg2da9iabigdaXaqaaiabd2gaTjabgkHiTiabigdaXaqdcqGHris5aaGcbaaabaGaeyypa0dabaGaemyBa0MaeiikaGIae8NSdiMaeyOeI0Iae8xSdeMaeiykaKIaey4kaSYaaabmaeaacqqGcbGqcqqGtbWucqGGOaakcqWGnbqtcqGGBbWwcqWGPbqAcqGGUaGlcqGGUaGlcqWGTbqBcqGGDbqxcqGGPaqkcqGHsislcqqGxbWvcqqGtbWucqGGOaakcqWGnbqtcqGGBbWwcqWGPbqAcqGGUaGlcqGGUaGlcqWGTbqBcqGGDbqxcqGGPaqkaSqaaiabdMgaPjabg2da9iabikdaYaqaaiabd2gaTbqdcqGHris5aaGcbaaabaGaeyypa0dabaGaemyBa0MaeiikaGIae8NSdiMaeyOeI0Iae8xSdeMaeiykaKIaey4kaSYaaabmaeaadaaeWaqaaiab=r7aKnaaBaaaleaacqWGQbGAaeqaaaqaaiabdQgaQjabg2da9iabdMgaPbqaaiabd2gaTbqdcqGHris5aaWcbaGaemyAaKMaeyypa0JaeGOmaidabaGaemyBa0ganiabggHiLdaakeaaaeaacqGH9aqpaeaacqWGTbqBcqGGOaakcqWFYoGycqGHsislcqWFXoqycqGGPaqkcqGHRaWkdaaeWaqaaiabcIcaOiabdMgaPjabgkHiTiabigdaXiabcMcaPiab=r7aKnaaBaaaleaacqWGPbqAaeqaaaqaaiabdMgaPjabg2da9iabikdaYaqaaiabd2gaTbqdcqGHris5aaaaaaa@C16D@

Since permutation of matrices induces a permutation of the sequence *δ*_2_,..., *δ*_*m*_, the value ∑i=2m(i−1)δi
 MathType@MTEF@5@5@+=feaafiart1ev1aaatCvAUfKttLearuWrP9MDH5MBPbIqV92AaeXatLxBI9gBaebbnrfifHhDYfgasaacPC6xNi=xH8viVGI8Gi=hEeeu0xXdbba9frFj0xb9qqpG0dXdb9aspeI8k8fiI+fsY=rqGqVepae9pg0db9vqaiVgFr0xfr=xfr=xc9adbaqaaeGacaGaaiaabeqaaeqabiWaaaGcbaWaaabmaeaacqGGOaakcqWGPbqAcqGHsislcqaIXaqmcqGGPaqkiiGacqWF0oazdaWgaaWcbaGaemyAaKgabeaaaeaacqWGPbqAcqGH9aqpcqaIYaGmaeaacqWGTbqBa0GaeyyeIuoaaaa@3A9D@ is minimal when *δ*_1 _≥ *δ*_2 _≥ ... ≥ *δ*_*m*_.

In the remaining of this paper, we shall always assume that the matrix *M *has been permuted so that it fulfills the condition on (*δ*_*i*_)_1≤*i*≤*m *_of Lemma 7. This is simply a pre-processing of the matrix that does not affect the course of the algorithms.

### Efficient algorithms for computing the P-value without error

We now come to the presentation of two exact algorithms, which is are the main algorithms of this paper. In Algorithms SCOREDISTRIBUTION and FASTPVALUE, the number of accessible scores plays an essential role in the time and space complexity. As mentioned in the Background section, this number can be as large as |Σ|^*m*^. In practice, it strongly depends on the involved matrix and on the way the score distribution is approximated by round matrices. The choice of the precision is critical. Algorithms SCOREDISTRIBUTION and FASTPVALUE should compromise between accuracy, with faithful approximation, and efficiency, with rough approximation.

To overcome this problem, we propose to define successive discretized score distributions with growing accuracy. The key idea is to take advantage of the shape of the score distribution *Q*, and to use small granularity values only in the portions of the distribution where it is required. This is a kind of selective zooming process. Discretized score distributions are built from round matrices.

**Definition 3 (Round matrix) ***Let M be a matrix of real coefficient values of length m and let ε be a positive real number. We denote M*_*ε *_*the round matrix deduced from M by rounding each value by ε:*

Mε(i,x)=ε⌊M(i,x)ε⌋
 MathType@MTEF@5@5@+=feaafiart1ev1aaatCvAUfKttLearuWrP9MDH5MBPbIqV92AaeXatLxBI9gBaebbnrfifHhDYfgasaacPC6xNi=xI8qiVKYPFjYdHaVhbbf9v8qqaqFr0xc9vqFj0dXdbba91qpepeI8k8fiI+fsY=rqGqVepae9pg0db9vqaiVgFr0xfr=xfr=xc9adbaqaaeGacaGaaiaabeqaaeqabiWaaaGcbaGaemyta00aaSbaaSqaaGGaciab=v7aLbqabaGccqGGOaakcqWGPbqAcqGGSaalcqWG4baEcqGGPaqkcqGH9aqpcqWF1oqzdaGbdaqcfayaamaalaaabaGaemyta0KaeiikaGIaemyAaKMaeiilaWIaemiEaGNaeiykaKcabaGae8xTdugaaaGccaGLWJVaay5+4daaaa@4521@

*ε is called the *granularity. *Given ε, we can define E, the *maximal error *induced by M*_*ε*_.

E=∑i=1mmax⁡{M(i,x)−Mε(i,x),x∈Σ}
 MathType@MTEF@5@5@+=feaafiart1ev1aaatCvAUfKttLearuWrP9MDH5MBPbIqV92AaeXatLxBI9gBaebbnrfifHhDYfgasaacPC6xNi=xI8qiVKYPFjYdHaVhbbf9v8qqaqFr0xc9vqFj0dXdbba91qpepeI8k8fiI+fsY=rqGqVepae9pg0db9vqaiVgFr0xfr=xfr=xc9adbaqaaeGacaGaaiaabeqaaeqabiWaaaGcbaGaemyrauKaeyypa0ZaaabCaeaacyGGTbqBcqGGHbqycqGG4baEcqGG7bWEcqWGnbqtcqGGOaakcqWGPbqAcqGGSaalcqWG4baEcqGGPaqkcqGHsislcqWGnbqtdaWgaaWcbaacciGae8xTdugabeaakiabcIcaOiabdMgaPjabcYcaSiabdIha4jabcMcaPiabcYcaSiabdIha4jabgIGiolabfo6atjabc2ha9bWcbaGaemyAaKMaeyypa0JaeGymaedabaGaemyBa0ganiabggHiLdaaaa@519A@

**Lemma 8 ***Let M be a matrix, ε the granularity, and E the maximal error associated. For each word u of *Σ^*m*^*, we have *0 ≤ Score(*u*, *M*) - Score(*u*, *M*_*ε*_) ≤ *E*.

**Proof**. This is a straightforward consequence of Definition 3 for *M*_*ε *_and *E*.

**Lemma 9 ***Let M, N and N' be three matrices of length m, E, E' be two non-negative real numbers, α, β be two scores such that α ≤ β, satisfying the following hypotheses:*

*(i) for each word u in *Σ^*m*^, Score(*u*, *N*) ≤ Score(*u*, *M*) ≤ Score(*u*, *N*) + *E*,

*(ii) for each word u in *Σ^*m*^, Score(*u*, *N'*) ≤ Score(*u*, *N*) ≤ Score(*u*, *M*) ≤ Score(*u*, *N'*) + *E'*,

*(iii) *P-value(*N*, *α *- *E*) = P-value(*N*, *α*),

*(iv) *P-value(*N'*, *β *- *E'*) = P-value(*N'*, *β*),

then

∑α≤t<βt accessibleQ(N,t)=∑α≤t<βt accessibleQ(M,t)
 MathType@MTEF@5@5@+=feaafiart1ev1aaatCvAUfKttLearuWrP9MDH5MBPbIqV92AaeXatLxBI9gBaebbnrfifHhDYfgasaacPC6xNi=xI8qiVKYPFjYdHaVhbbf9v8qqaqFr0xc9vqFj0dXdbba91qpepeI8k8fiI+fsY=rqGqVepae9pg0db9vqaiVgFr0xfr=xfr=xc9adbaqaaeGacaGaaiaabeqaaeqabiWaaaGcbaWaaabuaeaacqWGrbqucqGGOaakcqWGobGtcqGGSaalcqWG0baDcqGGPaqkaSqaauaabeqaceaaaeaaiiGacqWFXoqycqGHKjYOcqWG0baDcqGH8aapcqWFYoGyaeaacqWG0baDcqqGGaaicqWGHbqycqWGJbWycqWGJbWycqWGLbqzcqWGZbWCcqWGZbWCcqWGPbqAcqWGIbGycqWGSbaBcqWGLbqzaaaabeqdcqGHris5aOGaeyypa0ZaaabuaeaacqWGrbqucqGGOaakcqWGnbqtcqGGSaalcqWG0baDcqGGPaqkaSqaauaabeqaceaaaeaacqWFXoqycqGHKjYOcqWG0baDcqGH8aapcqWFYoGyaeaacqWG0baDcqqGGaaicqWGHbqycqWGJbWycqWGJbWycqWGLbqzcqWGZbWCcqWGZbWCcqWGPbqAcqWGIbGycqWGSbaBcqWGLbqzaaaabeqdcqGHris5aaaa@6C5D@

**Proof**. Let *u *be a string in Σ^*m*^. It is enough to establish that *α *≤ Score(*u*, *N*) <*β *if, and only if, *α *≤ Score(*u*, *M*) <*β*. The proof is divided into four parts.

- If *α *≤ Score(*u*, *N*), then *α *≤ Score(*u*, *M*): This is a consequence of Score(*u*, *N*) ≤ Score(*u*, *M*) in (i).

- If *α *≤ Score(*u*, *M*), then *α *≤ Score(*u*, *N*): By hypothesis (i) on *E*, *α *≤ Score(*u*, *M*) implies *α *- *E *≤ Score(*u*, *N*). Since P-value(*N*, *α *- *E*) = P-value(*N*, *α*) with (iii), it follows that *α *≤ Score(*u*, *N*).

- If Score(*u*, *N*) <*β*, then Score(*u*, *M*) <*β*: By hypothesis (ii), Score(*u*, *N*) <*β *implies that Score(*u*, *N'*) <*β*. According to (iv), this ensures that Score(*u*, *N'*) <*β *- *E'*, which with (ii) guarantees Score(*u*, *M*) <*β*

- If Score(*u*, *M*) <*β*, then Score(*u*, *N*) <*β*: This is a consequence of Score(*u*, *N*) ≤ Score(*u*, *M*) in (i).

What does this statement tell us ? It provides a sufficient condition for the distribution score *Q *computed with a round matrix to be valid for the initial matrix *M*. Assume that you can observe two plateaux ending respectively at *α *and *β *in the score distribution of *M*_*ε*_. Then the approximation of the total probability for the score range [*α*, *β*[obtained with the round matrix is indeed the exact probability. In other words, there is no need to use smaller granularity values in this region to improve the result.

#### From score to P-value

**Figure 5:**
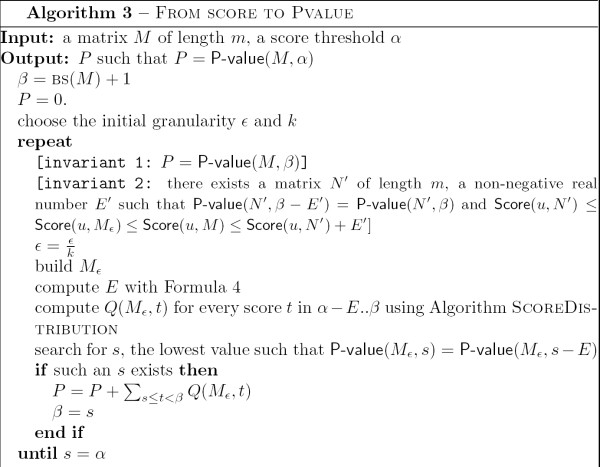
Algorithm From Score to P-value.

Lemma 9 is used through a stepwise algorithm to compute the P-value of a score threshold. Let *α *be the score for which we want to determine the associated P-value. We estimate the score distribution *Q *iteratively. For that, we consider a series of round matrices *M*_*ε *_for decreasing values of *ε*, and calculate successive values P-value (*M*_*ε*_, *α*). The efficiency of the method is guaranteed by two properties. First, we introduce a stop condition that allows us to stop as soon as it is guaranteed that the exact value of the P-value is reached. Second, we carefully select relevant portions of the score distribution for which the computation should go on. This tends to restrain the score range to inspect at each step. The algorithm is displayed in Figure 5.

The correctness of the algorithm comes from the two next Lemmas. The first Lemma establishes that the loop invariants hold.

**Lemma 10 ***Throughout Algorithm 3, the variables β and P satisfy the invariant relation P *= P-value(*M*, *β*).

**Proof**. This is a consequence of invariant 1 and invariant 2 in Algorithm 3. Both invariants are valid for initial conditions. When *P *= 0 and *β *= BS(*M*) + 1: P-value(*M*, BS(*M*) + 1) = 0. Regarding *N'*, choose *N' *= *M*_*ε*_.

There are two cases to consider for invariant 1.

- If *s *does not exist. *P *and *β *remain unchanged, so we still have *P *= P-value(*M*, *β*). Regarding invariant 2, if there exists such a matrix *N' *at the former step for *M*_*kε*_, then it is still suitable for *M*_*ε*_.

- If *s *actually exists. invariant 1 implies that *P *is updated to P-value(*M*, *β*) + ∑_*s*≤*t*<*β *_*Q*(*M*_*ε*_, *t*).

According to Lemma 9 and invariant 2, we have ∑_*s*≤*t*<*β *_*Q*(*M*_*ε*_, *t*) = ∑_*s*≤*t*<*β *_*Q*(*M*, *t*). Hence *P *= P-value(*M*, *s*). Since *β *is updated to *s*, it follows that *P *= P-value(*M*, *β*). Regarding invariant 2, take *N' *= *M*_*ε*_.

The second Lemma shows that when the stop condition is met, the final value of the variable *P *is indeed the expected result P-value(*M*, *α*).

**Lemma 11 ***At the end of Algorithm 3, P *= P-value(*M*, *α*).

**Proof**. When *s *= *α *- *E*, then *β *= *α*. According to Lemma 10, it implies *P *= P-value(*M*_*ε*_, *α*). Since the stop condition implies that P-value(*M*_*ε*_, *α *- *E*) = P-value(*M*_*ε*_, *α*), Lemma 9 ensures that P-value(*M*_*ε*_, *α*) = P-value(*M*, *α*).

#### From P-value to score

Similarly, Lemma 9 is used to design an algorithm to compute the score threshold associated to a given P-value. We first show that the score threshold obtained with a round matrix for a P-value gives some insight about the potential score interval for the initial matrix *M*.

**Lemma 12 ***Let M be a matrix, ε a granularity and E the maximal error associated. Given P*, 0 ≤ *P *≤ 1, *we have*

Threshold(*M*_*ε*_, *P*) ≤ Threshold(*M*, *P*) ≤ Threshold(*M*_*ε*_, *P*) + *E*

**Proof**. Let *β *= Threshold(*M*_*ε*_, *P*). According to Lemma 8, P-value(*M*_*ε*_, *β*) ≥ *P *implies P-value(*M*, *β*) ≥ *P*, which yields *β *≤ Threshold(*M*, *P*). So it remains to establish that Threshold(*M*, *P*) ≤ *β *+ *E*. If P-value(*M*, *β *+ *E*) = 0, then the highest accessible score for *M *is smaller than *β *+ *E*. In this case, the expected result is straightforward. Otherwise, there exists *β' *such that *β' *is the lowest accessible score for *M *that is strictly greater than *β *+ *E*. Since *s *→ P-value(*M*, *s*) is a decreasing function in *s*, we have to verify that P-value(*M*, *β'*) <*P *to complete the proof of the Lemma. Assume that P-value(*M*, *β'*) ≥ *P*. Let *γ *= min {Score(*u*, *M*_*ε*_)|*u *∈ Σ^*m *^∧ Score(*u*, *M*) ≥ *β'*}. On the one hand, the definition of *γ *implies that

P-value(*M*, *β'*) ≤ P-value(*M*_*ε*_, *γ*)

On the other hand, *γ *is an accessible score for *M*_*ε *_that satisfies *γ *≥ *β' *- *E *> *β*. By hypothesis of *β*, it follows that

P-value(*M*_*ε*_, *γ*) <*P*

Equations 5 and 6 contradict the assumption that P-value(*M*, *β'*) ≥ *P*. Thus P-value(*M*, *β'*) <*P*.

**Figure 6:**
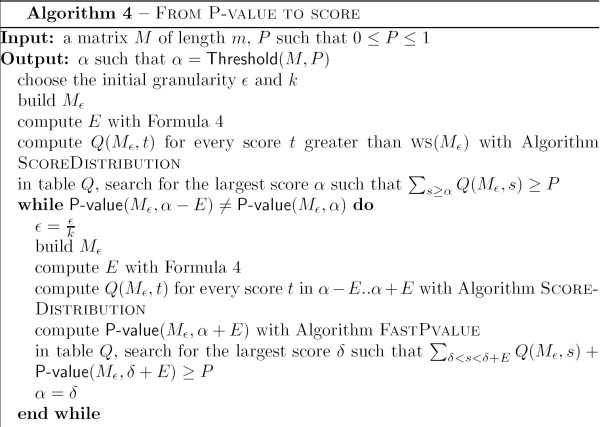
Algorithm From P-value to Score.

The sketch of the algorithm is as follows. Let *P *be the desired P-value. We compute iteratively the associated score threshold for successive decreasing values of *ε*. At each step, we use Lemma 12 to speed the calculation for the matrix *M*_*ε*_. This Lemma allows us to restrain the computation of the detailed score distribution *Q *to a small interval of length 2 × *E*. For the remaining of the distribution, we can use the FASTPVALUE algorithm. Lemma 13 ensures that when P-value(*M*_*ε*_, *α *- *E*) = P-value(*M*_*ε*_, *α*), then *α *is the required score value for *M*. The algorithm is displayed in more details in Figure 6.

**Lemma 13 ***Let M be a matrix, ε the granularity and E the maximal error associated. If *P-value(*M*_*ε*_, *α *- *E*) = P-value(*M*_*ε*_, *α*)*, then *P-value(*M*, *α*) = P-value(*M*_*ε*_, *α*).

**Proof**. This is a corollary of Lemma 9 with *M*_*ε *_in the role of *N *and *N'*, and BS(*M*) + *E *in the role of *β*.

## Experimental Results

The ideas presented in this paper have been incorporated in a software called TFM-PVALUE (TFM stands for *Transcription factor matrix*). The software is written in C++ and implements the FROM PVALUE TO SCORE and FROM SCORE TO PVALUE algorithms as described in Algorithms 5 and 6, together with permutated lookahead scoring. It is available for download at [17]. In the worst case, TFM-PVALUE does not improve the theoretical complexity of the score threshold problem. This was expected from the NP-hardness proof provided in the second section. Nevertheless, experimental results show considerable speedups in practice.

### Methods

We chose a multinomial background model with identically and independently distributed character symbols on the four letter alphabet {*A*, *C*, *G*, *T*} to conduct our experiments. The decreasing step (*k*) in the algorithm was set to 10 and the initial granularity (*ε*) was set to 0.1. The test set is made of the Jaspar database of transcription factor binding sites [1]. It contains 123 matrices, whose length ranges from 4 to 30. The matrices are transformed into log-ratio matrices following the technique given in [18]. For each P-value *P*, we report only results for matrices whose length is suitable for *P*: we requested that the probability of a single word is smaller than *P*. So a matrix of length *m *cannot not achieve a P-value smaller than 14m
 MathType@MTEF@5@5@+=feaafiart1ev1aaatCvAUfKttLearuWrP9MDH5MBPbIqV92AaeXatLxBI9gBaebbnrfifHhDYfgasaacPC6xNi=xH8viVGI8Gi=hEeeu0xXdbba9frFj0xb9qqpG0dXdb9aspeI8k8fiI+fsY=rqGqVepae9pg0db9vqaiVgFr0xfr=xfr=xc9adbaqaaeGacaGaaiaabeqaaeqabiWaaaGcbaqcfa4aaSaaaeaacqaIXaqmaeaacqaI0aandaahaaqabeaacqWGTbqBaaaaaaaa@2FDC@. For example, matrices of length 4 have not been considered for a P-value equal to 10^-3^, and matrices of length smaller than 10 have not be considered for a P-value equal to 10^-6^.

Experimental results are concerned with the error rate depending on the chosen granularity. To estimate the error made at a given granularity, we first computed *α*_*ε*_, the score threshold associated to the P-value with the round matrix *M*_*ε*_, and *a *the score threshold associated to the P-value with the original matrix *M*. We then denumerate the number of words whose score is between *α*_*ε *_and *α *for *M*. Concerning the time efficiency, all computation times were measured on a 2.33 GHz Intel Core 2 Duo processor with 2 Go of main memory under Mac OS 10.4.

Concerning FROM P-VALUE TO SCORE, We also compared our results with those of algorithm LAZYDISTRIBUTION described in [6]. To the best of our knowledge, this algorithm is the most efficient algorithm today to compute the score associated to a P-value. It uses the dynamic programming formulas of Equation 1 in a lazy way and takes advantage of permutated lookahead scoring as presented in the previous Section. We implemented it in C++, like TFM-PVALUE.

### Computation times for a given granularity

In this first experiment, we study the time performance of TFM-PVALUE compared to LAZYDISTRIBUTION when using the same approximation for the distribution score. So in both cases we use round matrices with the same granularity. To set a maximal granularity for TFM-PVALUE, we interrupt the loop of decreasing granularities and output the score threshold found at this granularity. We thus obtain exactly the same score threshold as LAZYDISTRIBUTION.

#### Granularity 10^-3^

**Figure 7:**
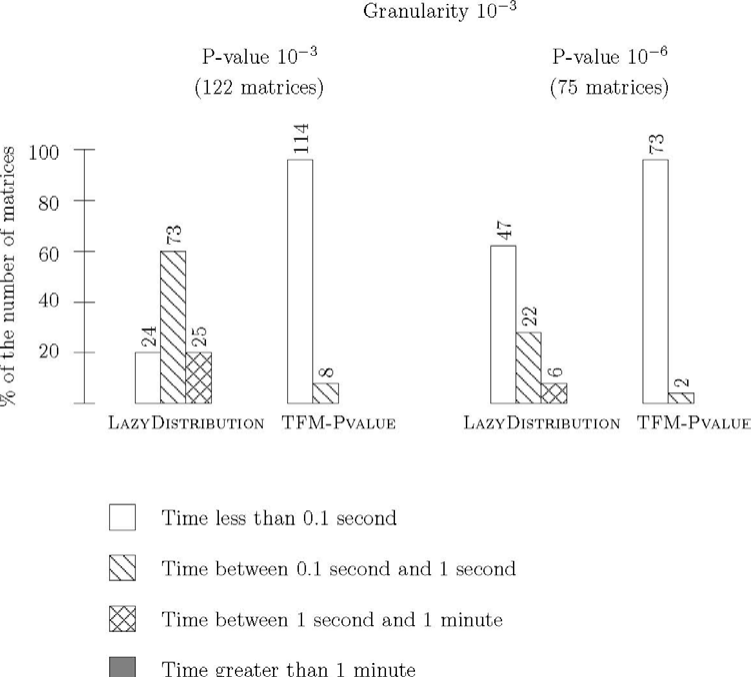
**Time efficiency for granularity 10^-3^**. We compare the running time for the computation of the score threshold associated to a given P-value for FROM P-VALUE TO SCORE and LAZYDISTRIBUTION onto the Jaspar matrices with a granularity set to 10^-3^. We choose two P-value levels: 10^-3 ^and 10^-6^. There are 122 matrices (resp. 75 matrices) that can achieve a P-value equal to 10^-3 ^(resp. 10^-6^). For each algorithm, we classified the matrices into four groups according to the time needed to complete the computation: less than 0.1 second, from 0.1 second to 1 second, from 1 second to 1 minute, and greater than 1 minute. The results are represented by a histogram with four bars. The height of each bar gives the percentage of matrices involved and the number at the top of each bar indicates the corresponding number of matrices.

We first chose a granularity of 10^-3 ^for the two algorithms and computed the score associated to P-values equal to 10^-3 ^and 10^-6 ^for each matrix of the Jaspar database (see Figure 7). The results show that TFM-PVALUE outperforms LAZYDISTRIBUTION in both cases. With the P-value set to 10^-3^, the average computation time is 0.64 second per matrix for LAZYDISTRIBUTION compared to 0.03 second for TFM-PVALUE. Considering each matrix individually, TFM-PVALUE is 61 times faster than LAZYDISTRIBUTION. With the P-value set to 10^-6^, the average computation time is 0.118 second per matrix for LAZYDISTRIBUTION and 0.019 second for TFM-PVALUE. Considering each matrix individually, TFM-PVALUE is 15 times faster than LAZYDISTRIBUTION.

#### Granularity 10^-6^

**Figure 8:**
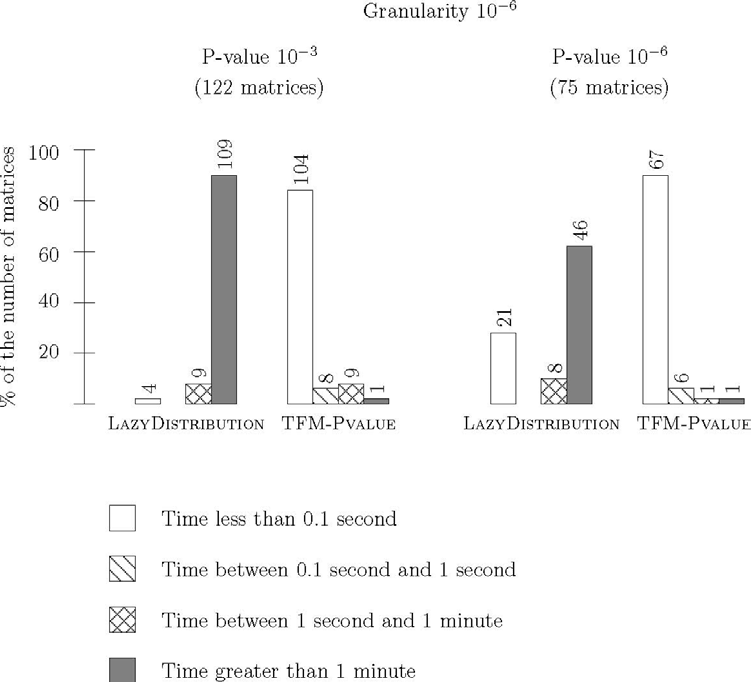
**Time efficiency for granularity 10^-6^**. We compare the computation time for the score associated to a P-value of 10^-3 ^and 10^-6 ^onto the Jaspar matrices when the granularity is set to 10^-6 ^for TFM-PVALUE and LAZYDISTRIBUTION. The histogram has the same meaning as in Figure 3.

We then repeated the same procedure as above with a smaller granularity, 10^-6 ^instead of 10^-3^. Results are reported in Figure 8. When the granularity decreases, the computation time of LAZYDISTRIBUTION dramatically increases. With the P-value set to 10^-3^, LAZYDISTRIBUTION needs a running time greater than one minute for 89 percent of the matrices (109 out of 122). TFM-Pvalue needs less than 0.1 second for 85 percent of the matrices (104 out of 122). With the P-value set to P-value = 10^-6^, LAZYDISTRIBUTION needs a computation time greater than 1 minute for 62 percent of matrices (47 out of 75). TFM-PVALUE needs less than 0.1 second for 89 percent of matrices (67 out of 75). Moreover, if we compare the histogram for TFM-PVALUE in Figure 8 with the histogram for LAZYDISTRIBUTION in Figure 7, it appears that TFM-PVALUE is still more efficient, whereas the granularity is a thousand fold larger. This demonstrates that we are able to provide more accurate results within the same amount of time. The same conclusion holds for the amount of memory needed to achieve the computation (data not shown).

### Ability to compute accurate thresholds

**Figure 9:**
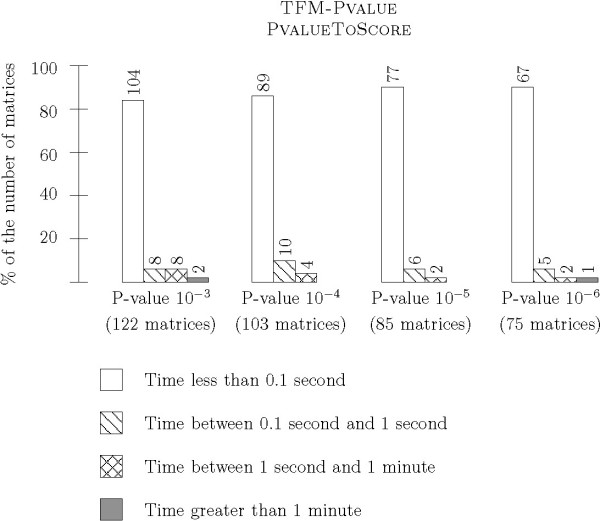
**Runtime of TFM-Pvalue – From P-value to Score without any granularity bound**. This histogram shows time measurements for the P-VALUE TO SCORE algorithm without any granularity bound. The algorithm stops when it is guaranteed to find the exact P-value, without error. We ran tests on a variety of P-value parameters: 10^-3^, 10^-4^, 10^-5^, and 10^-6^. As previously, we report the proportion of matrices for which the runtime was less then 0.1 second, between 0.1 second and 1 second, between 1 second and 1 minute and greater than 1 minute.

**Figure 10:**
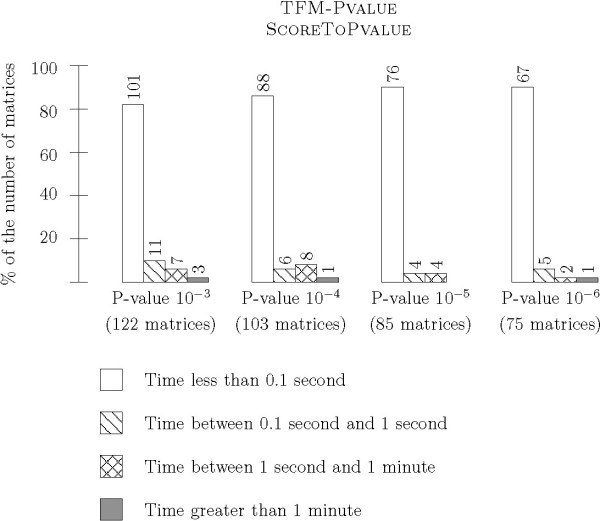
**Runtime of TFM-Pvalue – From Score to P-value without any granularity bound**. This histogram shows time measurements for the SCORE TO PVALUE algorithm without any granularity bound. We chose initial scores corresponding to a P-value of 10^-3^, 10^-4^, 10^-5 ^and 10^-6^.

In the second series of experiments, we tested the ability of TFM-PVALUE to get exact score thresholds within a reasonable amount of time. We ran FROM P-VALUE TO SCORE and FROM SCORE TO P-VALUE without setting a maximal granularity so that the algorithms stop when they reach the correct result. We tried several P-values, from 10^-3 ^to 10^-6^, for all matrices of suitable length. Runtime is reported in Figure 9 for FROM P-VALUE TO SCORE and in Figure 10 for FROM SCORE TO P-VALUE. Regarding FROM SCORE TO P-VALUE, the time required to compute the score thresholds remains very small for a large majority of matrices: less than 0.01 second for 253 out of the 383 computations for P-values from 10^-3 ^to 10^-6^, and less than 0.1 second for 337 computations. As expected, results for FROM SCORE TO P-VALUE are very similar: less than 0.01 second for 332 out of the 383 computations for P-values from 10^-3 ^to 10^-6^, and less than 0.1 second for 358 computations.

We display in Table 2 the value of the granularity required to guarantee an exact score threshold in function of the range of P-values with FROM P-VALUE TO SCORE. The results show that a granularity lower than or equal to 10^-4 ^is often needed: more than 63 percent. It is interesting to remark that the granularity does not directly depend on the length of the matrices. In fact, it depends of the shape and density of the score distribution around the score corresponding to the P-value required. Nevertheless, as the size of the matrix increases, the number of words greater than a score grows for a given P-value and hence the granularity needs to be lower. To illustrate this, all matrices with length less than or equal to 9 need a granularity ranging from 10^-1 ^and 10^-5^, whereas all matrices with length greater than or equal to 13 need a granularity ranging from 10^-4 ^and 10^-9^.

**Table 2:** Granularity required for accurate computation with From P-value to Score. This table indicates the granularity value that is required for FROM P-VALUE TO SCORE to compute the accurate score threshold without any error. Each row of the table corresponds to a P-value: 10^-3^, 10^-4^, 10^-5^, and 10^-6^. Each cell gives the percentage of matrices for which FROM P-VALUE TO SCORE ends at the granularity of the corresponding column. For example, 52.4% matrices need a granularity larger than or equal to 10^-3 ^when computing threshold for P-value 10^-5^.

	Granularity								
P-value	1e-1	1e-2	1e-3	1e-4	1e-5	1e-6	1e-7	1e-8	1e-9
1e-3	9	22.9	39.3	63.1	77.8	88.5	91.8	95.1	100
1e-4	7.7	20.2	49	70.2	85.6	92.3	97.1	99	100
1e-5	1.2	25.6	52.4	76.8	88.4	94.2	96.5	96.5	100
1e-6	5.4	42.7	66.7	82.7	94.7	96	98.7	98.7	100

**Figure 11:**
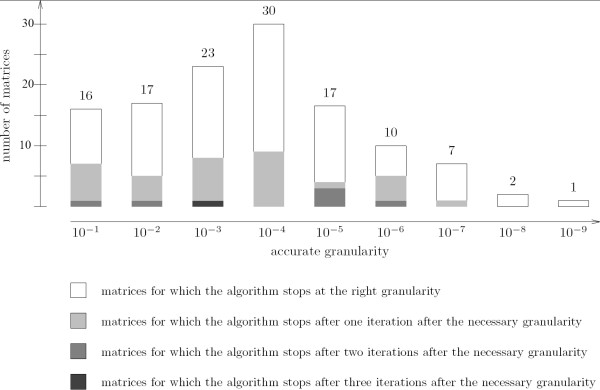
**Granularity required for accurate computation with From Score to P-value**. This figure compares the theoretical necessary granularity and the granularity reached by FROM SCORE TO P-VALUE. For example, granularity 10^-2 ^is necessary for 17 matrices. It means that the round matrix with granularity 10^-1 ^gives a wrong P-value, whereas the round matrix with granularity 10^-2 ^gives an accurate P-value. Amongst these 17 matrices, FROM SCORE TO P-VALUE stops at 10^-2 ^for 11 matrices, performs one supplementary step at 10^-3 ^for 5 matrices, and two supplementary steps, at 10^-3 ^and 10^-4^, for one matrix.

We also evaluated the behavior of FROM SCORE TO P-VALUE. For each matrix, for a given score threshold corresponding to a P-value of 10^-3^, we computed the largest granularity necessary to obtain an accurate result with a round matrix. Results are summarized in Figure 11. We then compared this granularity with the granularity found with FROM SCORE TO P-VALUE. In more than 60 percent of matrices, FROM SCORE TO P-VALUE stops as soon as it is possible, with no extra iteration. In more than 90 percent of matrices, it is able to conclude with at most one supplementary step.

## Discussion and Conclusion

We performed an extensive analysis of the computation of P-values for matrices. We gave a simple proof that the *From P-value to score *and *From Score to P-value *problems are NP-hard. We then presented two algorithms to solve them efficiently and accurately for real-life examples. As the problem is intrinsically difficult, the worst complexity is not changed and then some matrices may require large computation time and memory. Fortunately, our experiments show that this arises only in very few cases. Our algorithms can be of interest for at least two tasks. First, they can be exploited to obtain significantly faster algorithms than existing ones when a loss of precision is allowed. Indeed, for a same computation time and amount of memory, our algorithms perform better than existing ones. This allows to avoid pre-computation of scores associated to fixed P-values as done in some software programs [16], and to compute the desired P-value on the fly, as specified by the user. Secondly, the algorithms can be used where it is needed to compute a score threshold with high precision, with arbitrary low granularity, in a reasonable amount of space and time. We provided thus a significant improvement to compute scores and P-values with high accuracy.

When running experiments on Jaspar database, we chose a value for *k*, the decreasing step for successive granularities, equal to 10. A different value may be selected. With a lower decreasing step value, the algorithms stop with more accurate granularity and so may avoid useless computations. But this leads to more iterations and then globally to a higher runtime. With a larger decreasing step value, there are less iterations and then the global runtime is lowered. But choosing a very large decreasing step value (more than 10^3 ^for example) amounts to compute almost the complete score distribution and the algorithms become inefficient because they do not take advantage of the reduction of the score range for which exact P-values are computed. As the algorithms are mainly based onto the computation of accessible scores, the memory required is almost the same independently of the decreasing step value (until the value is not very large).

When we allowed for some error, such as in the first experiment, this implicitly amounts to calculate the exact score distribution, and thus the exact P-value, for the round matrix as described in Definition 3. One can choose an alternative rounding construction for the initial matrix, such as ε×⌊M(i,x)ε+0.5⌋
 MathType@MTEF@5@5@+=feaafiart1ev1aaatCvAUfKttLearuWrP9MDH5MBPbIqV92AaeXatLxBI9gBaebbnrfifHhDYfgasaacPC6xNi=xH8viVGI8Gi=hEeeu0xXdbba9frFj0xb9qqpG0dXdb9aspeI8k8fiI+fsY=rqGqVepae9pg0db9vqaiVgFr0xfr=xfr=xc9adbaqaaeGacaGaaiaabeqaaeqabiWaaaGcbaacciGae8xTduMaey41aq7aayWaaeaajuaGdaWcaaqaaiabd2eanjabcIcaOiabdMgaPjabcYcaSiabdIha4jabcMcaPaqaaiab=v7aLbaakiabgUcaRiabicdaWiabc6caUiabiwda1aGaayj84laawUp+aaaa@412F@, before running TFM-PVALUE. This leaves the course of the algorithms unchanged.

Finally, in the paper, we assumed that the background model is provided with a multinomial model. All results, except permutated lookahead scoring, can be extended to more sophisticate random sources, such as Markov models [19]. The consequence is an increasing of the computation time by a factor |Σ|^*n*^, where *n *is the order of the Markov model. But the optimization based on successive decreasing granularities still holds.

## Authors' contributions

All authors equally contributed to this paper. All authors read and approved the final manuscript.
